# In Memoriam

**Published:** 1881-01

**Authors:** Ad. Lippe

**Affiliations:** Phila.


### IN MEMORIAM.

BY AD. LIPPE, M. D., PHILA.

The late Constantine Hering, M.D., devoted his long life to the promulgation and development of the new healing art, called by its founder, Samuel Hahnemann, "Homœopathy." Dr. Hering furnished more valuable and reliable additions to our *Materia Medica* than all other physicians, since Hahnemann gave us his "Materia Medica Pura," and his "Chronic Diseases." Thereby he increased our ability to apply our immutable law of cure, with certainty and precision, to a larger number of diseased conditions.

The offerings Dr. Hering laid on the altar of the newly erected temple of our healing art were thankfully received by his contemporaries. They will forever be remembered as an evidence of the untiring devotion and industry of our late colleague to the cause he so earnestly and so sincerely espoused. He left us these fruits of his labors, but he left us much more to remember him by:—his example; his devotion to principle; his fidelity to the teachings of the Master; his steadfastness of character; and, last but not least, his late act in signing the Declaration of Principles. His very last admonition will forever endear his memory to those who honored him in life.

In memory of the father of Homœopathy in this country, to defend the principles he advocated and the parting admonitions he gave us, we found this Journal. We shall endeavor to show the correctness of the principles which he and others have declared to be essential to Homœopathy, and also the absolute necessity of adhering to the strict inductive method of Hahnemann. The life and works of our late co-laborer show that he fully deserves that highest title among medical men; a title declared by Samuel Hahnemann, himself, to be the most honorable a true physician could deserve, that of the "Homœopathic Physician."

Dr. Hering uttered, in the last paper he addressed to the profession, a warning admonition to be found on page 31 of the *North American Journal of Homœopathy*, August 1880. He there says: "If our school ever gives up the strict inductive method of Hahnemann we are lost, and deserve to be mentioned only as a caricature in the history of medicine." The provocation for this sentence was a paper on Apis Mel., published by Dr. Goullon, Jr., of Weimar, Germany. Dr. Goullon showed that he was really ignorant of his subject, that he knew nothing of this valuable American remedy, published by Dr. Hering in his great work, "The American Medicines Proved," 1857. Ignorant of the remedy, and setting aside the strict inductive method of Hahnemann, he necessarily failed in obtaining favorable clinical results. Hence he began a tirade against Apis Mel., and against such men as Dr. Wolf, of Berlin, who had communicated the great healing powers of this American remedy.

The last sentence of this paper by Dr. Hering, reads, "One can not be purified by defiling others." That is just what Dr. Goullon tried to do. Because *he* did not obtain the same results, that others had frequently accomplished, all these men were by him defiled. Had Dr. Goullon used the same diligence as others had done, in becoming fully acquainted with the newly proved remedy, had he followed the strict inductive method of Hahnemann, he would not have committed the fatal error of attempting to purify himself by defiling others. Dr. Goullon was but one of many who have given up the strict inductive method of Hahnemann, and who, failing to obtain such results as are promised to those who make the experiment exactly as Hahnemann showed to be necessary—*absolutely* necessary—and when so performed are always obtained, invariably attack the method they *never* followed. Should then, as Dr. Hering said, our school give up this strict inductive method of Hahnemann, we are lost. It is obvious that Dr. Hering had fears that such a calamity might take place in the future and it is also obvious that he saw clearly how large a number of professing Homœopathists were led astray by just such men as Dr. Goullon.

Let us now examine the present position of our school, as observed by Dr. Hering, and then point out the remedy which would prevent the calamity which he thinks will befall us if the proper precautions are not taken in due time.

Ever since Hahnemann published his great text book "The Organon of the Healing Art," there have been men who could not appreciate the great and astonishing cures made by Homœopathists. It would have been but proper and right for every one who hoped to accomplish just such results as he had seen follow the practice of the healing art, as revealed and taught in that great master work of the founder of our school, to fully acquaint himself with the logically developed truths and methods of this work before he attempted to make the experiment. That such was not the case, even during Hahnemann's days, is very evident from his frequent lamentations over the perversion of his teachings, which are to be found in great numbers in all his writings and, even to the last, in his preface to "Arsenicum." In direct proportion, as the essential and fundamental declarations of the principles of our healing art were rejected, the necessary failures followed. These increasing disappointments led to further departures. Trinks and others first conceived the plan by which our Materia Medica could be made more acceptable to the materialists; this plan was to give pathological conditions and names of diseases amenable to certain remedies. It was this growing desire to give our school a scientific basis which caused a return to hypotheses concerning really existing forms of diseases, thereby expecting to facilitate the clinical application of the law of the similars. It also brought us back to generalization instead of the more difficult and pains-taking plan of individualizing each and every case of sickness. By what method of reasoning any one could possibly arrive at the conclusion that the law of the similars was applicable to fixed forms of diseases is utterly incomprehensible to any person fully familiar with Hahnemann's methods. There might be a plausible excuse for this fatal error if it were shown that systematized forms of diseases affect all sick persons alike; but it was shown by Hahnemann, and can be seen by any observing healer, that the contrary is the case; that each sick person complains of different symptoms. And as we are taught that the extraordinary and peculiar symptoms, in each case of disease are the most important indications for the choice of the curative Homœopathic remedy, it becomes obvious that this very first departure from our curative method is preposterous.

The earliest observations made by Hahnemann, clearly showing the necessity of individualizing, have since been confirmed by every true healer. Let it never be forgotten how these early observations were made. When Hahnemann translated Cullen's remark on Cinchona, viz., that it was a specific remedy for intermittent fever because it was the most bitter and aromatic medicine, he paused. That short sentence was so full of erroneous statements that Hahnemann, who knew that Cinchona cured some cases of intermittent fever and did not show any curative or other powers in other cases, that it was not even the most bitter and aromatic medicine, concluded, at that memorable midnight hour, to clear up this therapeutic chaos. Cullen was then an authority, but he failed to reason rightly. Cinchona officinalis was the first medicine Hahnemann proved on himself. That proving convinced this great observer and acute reasoner that the sick-making powers of a drug, revealed to us its healing powers. The characteristic symptoms of Cinchona were soon observed, and we learned under what peculiar indications it would cure certain forms of intermittent fever. The observations then made are just as valuable to-day, will be as valuable for all time to come; as are now, and ever will be the inductive reasoning to which this and other observations led this indefatigable philosopher. Each medicine was further shown to posess its own peculiar sick-making and health-restoring properties, in intermittent fevers as well as in all other forms of diseases.

The desire to find specific medicines for specific forms of diseases, made by a class of men calling themselves Homœopaths in general, but also in particular, rational Homœopaths and specificists, was a violation of that inductive method which led Hahnemann to other, and always corroborating, experiments; and finally, to the development of Homœopathy as we find this healing art taught in his "Organon."

As a foregone conclusion these rational Homœopaths failed to cure, and so gradually fell into greater errors. In order to produce some results from the application of so-called Homœopathic medicines, they were compelled to increase the dose, and ended by finally denying the efficacy of such doses as Hahnemann and his followers cured the sick with. It was in this manner that at last these men, who already had made a caricature of Homœopathy, raised a false issue, making the posological question the distinguishing line between those who followed Hahnemann's methods and cured the sick with potentized drugs, and those who abandoned his methods, and on that account had to resort to cruder doses. Even to this day men, taking advantage of the ignorance of the masses, do not hesitate for a moment to divide the Homœopathists into high and low potency men. This distinction, this issue, has been shown to be erroneous and false, time and again. For the present, we will not further dwell on the motives of the men who have committed themselves to this fatal error. The true distinction between Homœopathists is to be found in the fact that some have accepted Hahnemann's inductive method, while others attempt to practice a caricature of Homœopathy, setting aside this inductive method and with it, logically, all the other methods of Hahnemann.

These differences existed in Hahnemann's day, but they have become progressively wider, year by year. The caricatures to which these widening differences led have now become numerous. The Materia Medica was first caricatured by the so-called "Pharmaco-dynamics;" of late a new work—a superlative caricature—is making its debut in its first volume, called "Materia Medica and Therapeutics, arranged upon a Physiological and Pathological Basis."* What reason there may be for this attempt to base our Materia Medica upon physiology and pathology, we can not see. Physiological and pathological knowledge are necessary to the true healer; for this knowledge will assist him in the examination of the sick, also in finding symptoms he otherwise would overlook. It will assist him in the classification of the symptoms of the sick, will assist him in ordering a proper regime for the sick. But when the true healer, in prescribing, seeks the similar remedy, he compares the symptoms of the patient, carefully arranged, with those of our proved drugs; and when a drug is found similar in all respects to the symptoms of his case, he knows he has his certain curative agent. The true healer does not neglect, in this comparison, the mental symptoms (of which physiology and pathology, as these collateral branches *now* exist, can take no cognizance); nor does he fail to note the most peculiar and extraordinary symptoms of the patient (not necessarily found always under the disease present), for these are to him most important.

The author of the "Pharmaco-dynamics" now asserts that Hahnemann was "led in numerous instances to put down as pathogenetic, effects which were, obviously due to disease, or occasional causes." This mere assertion, unsustained by proofs, fails to show any reason why we should *distrust* Hahnemann and *trust* the man who makes this bold, unproved assertion. This learned gentleman says further, in a lecture on Homœopathy, entitled "What It Is." "There are two distinct ways of

* This work will be fully reviewed before long.

applying the method of Hahnemann among those who adopt it, both finding origin in Hahnemann himself—one diverging under the attraction of the light of modern science, the other prolonging his own line of advances into regions undreamt of by him." How can there be a divergence from Hahnemann's methods under the attraction of the light of modern science? Every observing and thinking medical man will by the light of modern science become more and more convinced of the correctness of Hahnemann's strict inductive method. The fact is there are those who are misled by modern mock-science, and there are those who are not so misled but still follow Hahneman's "line of advances," *i. e.* his strict inductive methods; that the former class will reach regions not dreamt of by that great philosopher is to be expected. If our learned friend believes that physiology and pathology have become exact sciences and that we should therefore re-model our Materia Medica and apply our law of cure under the guidance of these exact sciences, he is "*misled.*" What Hahnemann said about these collateral branches of medical knowledge in 1833* is just as true in 1881, and it will be so to the end of time.

What remedy can we then apply to promote the universal acceptance of Hahnemann's inductive method? It becomes incumbent upon those who heed Dr. Hering's last admonition to show by the light of modern science, that the fundamental principles on which Hahnemann based his new Healing Art are correct, and are sustained by every new step of modern science; this is the only remedy left us. The comparative results of the practical application of Hahnemann's method have been shown, by his strict followers; the very frequent interrogations directed to men who have from time to time attempted to introduce new departures, have never been answered. The revelations made by Prof. D. Gustav Jaeger, of Stuttgart, will soon be published to show how modern science as applied by a learned man, not belonging to our school of medicine, comes

* "Hahnemann's Organon of the Healing-Art." 5th Edition.

unsolicited to our aid at this time. In future papers we shall endeavor to show how modern science affirms our fundamental principles.

